# Collaborative servitization in service-oriented company: The case study of telco company

**DOI:** 10.1371/journal.pone.0302943

**Published:** 2024-05-15

**Authors:** Jovana Mihailović, Biljana Stošić, Radul Milutinović

**Affiliations:** 1 ICT Product Development, Belgrade, Serbia; 2 Department of Technology, Innovations and Development Management, Faculty of Organizational Sciences, University of Belgrade, Belgrade, Serbia; Zhejiang University of Technology, CHINA

## Abstract

There is a growing interest in the subject of product-service system (PSS) and collaborative servitization in academia and practice. However, the focus is on exploring the growth of manufacturing companies without specifically analyzing the growth of service companies in applying PSS. There are companies, especially in the telco industry, that expand their service business to complex bundles of products and services. The paper investigates PSS in the service company and the role of collaboration in different PSS development phases: idea generation, development and *go to market* phase. The study adopted case-based research conducted in international Telco organization. The research demonstrates how a company creates and commercializes integrated packets of products and services, it identifies partners company works with and the benefits and challenges of their cooperation. The study addresses collaboration with customers and identifies five different customer profiles according to their openness to participate in the development of PSS. The study highlights the importance of (1) collaboration models, (2) customer involvement, and (3) strategic focus in successful application of collaborative servitization. The findings complement the literature for collaborative servitization and offer concrete input for companies in terms of how to better organize business, profit from collaboration models and gain market advantage.

## 1. Introduction

In recent years, the subject of product-service systems (PSS) has gained significant attention in academic circles. PSS can be described as a combination of products and services bundled in a way to better fit customer needs and offered as a single solution to customers [1]. This approach to business aims to meet customer needs more effectively by providing more comprehensive and tailored solutions. Nowadays organizations’ goal is to deliver performance, more than a product to customers.

Most researchers have analyzed manufacturing companies and cases when services are added to the products [2–4]. Servitization, as a path toward PSS, can also happen in service companies, when products are added to services [5, 6]. Additionally, innovation in the domain of services is more complicated [7] and observing PSS in setting where products were added to services is challenging task [8].

An example from the telecommunication industry of integrating products into services is that of mobile communication providers giving a free mobile phone handset to customers who sign up for a service [9]. Nowadays, new technologies, especially 5G, represent an opportunity for telco operators to expand business and create new integrated packages of product and services from the field of IoT, security, cloud technologies [10, 11].

The competition in the field of information and communication technologies (ICT) is very intensive and if mobile operators want to be competitive in the market, they need new business models [12–15]. Servitization as a strategy in combination with collaboration models can be an opportunity for operators to succeed [16]. Mobile operators lack knowledge and experience of how to design and develop PSS, especially considering that they now compete with rivals such as IT companies, device manufacturers, platform providers, etc. Hence, mobile operators are a suitable environment to observe the collaboration practices and application of PSS in a service company.

With this study authors focus on service companies and investigate the following:

1. How companies can benefit from collaboration models in different phases of PSS development (idea generation, development and go to market phase) and what are the challenges—The link between the PSS and collaboration was previously investigated by several authors, however their research were focused on manufacturing companies that added services on top of their products [17–20].
2. The importance of customer participation in the development of PSS—Customer involvement in PSS development is investigated as an isolated topic in literature [1, 9, 21]. Results obtained from manufacturing industry mainly showed positive influence of customer participation in PSS development [22, 23], but there are also examples investigating negative side of close collaboration with customers [20, 24].
3. The strong strategic focus toward collaboration models in PSS development—Literature findings regarding the strategic aspect for implementing servitization through collaboration models have shown positive outcome (longer and improved customer relationship, competitive advantage, promotion toward sustainability) as well as negative outcome (increased additional costs, difficulties in coordinating multiple actors) [25–27].

Based on results obtained from the manufacturing industry and followed by a case study conducted in mobile operator company, it is argued that collaboration is very important in successful application of PSS in service-oriented companies.

Paper is structured in the following way: Section 2 represents the foundation of the research with literature review of servitization, different concepts of PSS and collaboration practices. The research methodology is described in Section 3. Section 4 comprises results of a case study. Discussion about potential implication derived from the case study and findings of the paper are presented in Section 5. Section 6 concludes the research with theoretical and managerial implications, as well as suggestions for future studies.

## 2. Research context

### 2.1. Collaborative servitization

PSS is a topic widely studied in academic literature [2, 28, 29]. The idea behind PSS is to create value for customers beyond the product itself, such as providing installation, maintenance, support, training, updates, and many other services. There are many interpretations of PSS concept in literature, some of them are: an integrated bundle of product and services; a marketable set of products and services; a business model; a business innovation strategy; a functional solution; integrated combination of products and services; customization and integration of products and services, their development, post development customer support [30].

For product-oriented companies, PSS offers growth of business by adding services to products, while for service-oriented companies it expands business by adding hardware [31]. In simple terms, a transition toward PSS can be defined as servitization [32]. Servitization is the innovation of organizations capabilities and processes to better create mutual value through a shift from selling products to selling PSSs [33] and adopting service dominant logic can be considered business model innovation [34, 35]. Servitization provides opportunities for companies in the sense of gaining competitive advantage, improving quality of customer relationship, expanding portfolio and creating new sources of income [36, 37]. To successfully implement PSS, companies should distinguish between PSS design (development) phase (conceptual phase [38] and detailed design [39]) and from *go to market* (deployment) phase because they differ both in terms of investments, benefits, and challenges [40].

To describe the process of servitization and PSS, one can observe the changes in characteristic features [16, 41]: ownership, payment model, location of operation, single or multiple customer operation, maintenance. It depends on the company’s strategy, how to combine the features and how the package will be offered to customers. Combination of features can be interpreted through business model. [42] defined 3 main types of PSS business models according to the ownership of the solution and the payment model: product-oriented PSS, use-oriented PSS and result-oriented PSS. The relationship between the supplier and customers is more interactive, open and complex in result-oriented PSS than in product-oriented PSS [43].

**Fig 1:**
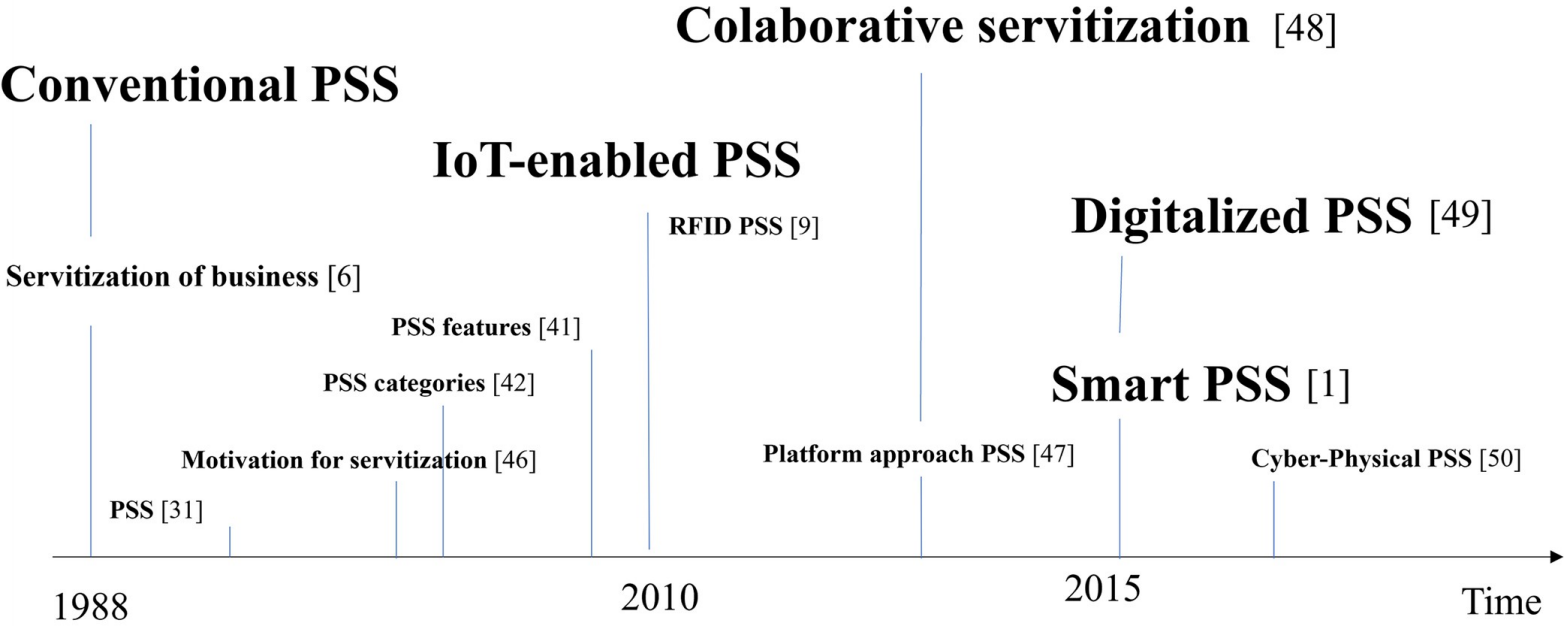
Different concepts of PSS and servitization and their first appearance in time [1, 6, 9, 31, 41, 42, 46–50].

Servitization is a long and continuous process [2] and the advancement of ICT brings out PSS to a higher level, by providing more service functionalities to customers [5, 19, 23]. For instance, sensors and cloud technologies are enabling data monitoring, storage, and post processing of data while data is used for creating new services. Technologies that are the biggest enablers of servitization are for example: IoT, predictive analytics; remote communications; consumption monitoring [44, 45]. Since the first appearance of term servitization in 1988 in [6] depending on context and technology that relies behind the PSS, different servitization concepts have been defined as presented in Fig 1.

It is extremely difficult for an enterprise to possess all relevant capabilities and resources for servitization due to its capacity’s limitations [51]. This implies that companies need to collaborate with partners and open their business models so as to deliver those services effectively and efficiently [20] and to capture value from it [52]. Hence, collaboration plays a key role in servitization [53–55]. Even though the term collaborative servitization, as an approach to servitization in which companies work together to create new PSS, was first mentioned in literature in 2014 [48], the field was not deeply investigated, and lately there is an increasing interest in this topic [27]. It was shown that collaborative partnerships, as a servitization strategy, increase performance outcomes, such as profit and end customer satisfaction [19, 53], however it also increases uncertainty and threat of failure due to third party dependency [20]. Collaborative servitization innovation is defined as a cooperative initiative to create and implement innovative solutions with simultaneous innovation of technology and service components [8, 54].

Existing studies mainly focus on single cooperative partners, but there is a lack of research on the mechanisms of multiple partners working together and more microlevel studies are needed concentrating on individual, group, or project levels [56]. One of the collaboration strategies organizations can implement to respond to customer needs quickly and efficiently, is to create a pool of partners. When customer requirement appears, companies can select a solution that can best respond to the actual use case [57]. [17] demonstrated that collaboration with partners influences (positively) service offerings, but it doesn’t impact the sale of solution. They also pointed out that collaboration activities of manufacturers depend on product complexity, export rate and cultural differences (size, sector, and country of a manufacturing company). Additionally, integration of partners and their competencies demands a certain maturity to provide market value [8] and successful functioning of network of providers depends on well-developed organizational readiness [58].

Close collaboration with customers contributes to effective servitization outcomes [8] and reduction of operational risk [59]. More than just targeting customer needs when designing solutions, solutions could be adaptable during their use to accommodate evolving customer needs [45]. Servitization and collaboration with customers was investigated in the manufacturing industry and have shown positive results in creating tailored solutions and capturing value for both customers and supplier [22, 23]. There are also examples investigating the negative side of close collaboration between provider and customers that can result in unrealized solutions due to lack of capabilities, unrealistic expectations, and poor cooperation [24] or customers might have limited perspective [60]. Nevertheless, by cooperating with customers manufacturers can also help them understand benefits and the value that comes out of new services [61].

Strategic planning and arrangements toward implementing servitization through collaboration models is very important [62]. It is a strategic decision if company will outsource missing competences, and accordingly how much they will rely on partners [63]. As shown in literature, companies are implementing new business models, going through transformation, managing new roles, and that can have positive outcomes (longer and improved customer relationship, competitive advantage, promotion toward sustainability) as well as negative outcomes (increased additional costs, difficulties in coordinating multiple actors) [25–27]. Additionally, strategic partnerships can strengthen a position on the market and create external service identity [63].

### 2.2. Servitization and telco companies

The telecommunication industry is undergoing continuous evolution due to growing need for faster data rates, external market competition and regulatory pressure and rising societal expectations. Back in the early 90s, 2G network was set up to support voice and short messaging services. 3G was introduced with the purpose of providing internet connectivity with very modest speed; afterwards, in 2010s 4G, a completely IP based system, had higher data speeds, quality and capacity services. In 5G focus is to deliver high-quality services to all devices, while future 6G are usually mentioned together with advanced technologies such as artificial intelligence, blockchain, etc. [64].

Technological changes can negatively impact the performance of large telco firms, but it is also an opportunity for operators to redefine their position on the market and improve business [65]. Disruptive change in telco industry impacts mobile operators and their business forcing them to succeed in three domains: infrastructure management (to follow the technology trend and enable connectivity services operators are well known for), to create and sell new products and services and customer relationship management (identify which business model suits customer the best) [13].

With the development of embedded SIM card (eSIM), operators faced new challenges and opportunities. ESIM means flexible connectivity, that customer can change operators without physically having to change the SIM card [66]. Changing operator is activity that is programmable remotely via software, and local profile switching for IoT devices can be realised in fast and energy efficient way [67]. ESIM might also mean higher customer churn, increased competition within the digital ecosystem, decline in roaming revenue, decline in average revenue per user, hence new attractive business models are needed to retain customers and increase the value they bring to operator [66, 68].

Nowadays, new technologies, especially 5G, represent an opportunity for mobile operators to expand business and create new integrated packages of product and services from the field of IoT, security, cloud technologies [10, 11]. This statement is supported by statistical data showing that the number of IoT devices in 2020 was 15.1 billion and it is expected to grow to more than 29 billion in 2030 [69].

In highly competitive environment like telecommunications, rivals are not just other operators, but many other companies such as system integrators, device manufacturers, platform providers, IT companies [70]. To establish a unique market position and strengthen its competitiveness on the market, telco operators should use distinct approaches and new business models [12, 14, 71, 72]. Servitization is one possible way [16]. Additionally, since operators lack skills, they need strategies to find partners and end-users [73] and hence there is a need to examine collaborative servitization in telco operator environment.

## 3. Research methodology

The methodology used for this research is a qualitative methodology based on a multiple-case study approach. Qualitative case study research was the method mostly used in similar research that can be found in literature and have given quite good results in regard to understanding collaborative servitization, the impact of customer involvement and strategic focus toward successful PSS development [22, 23, 57–59].

**Fig 2:**
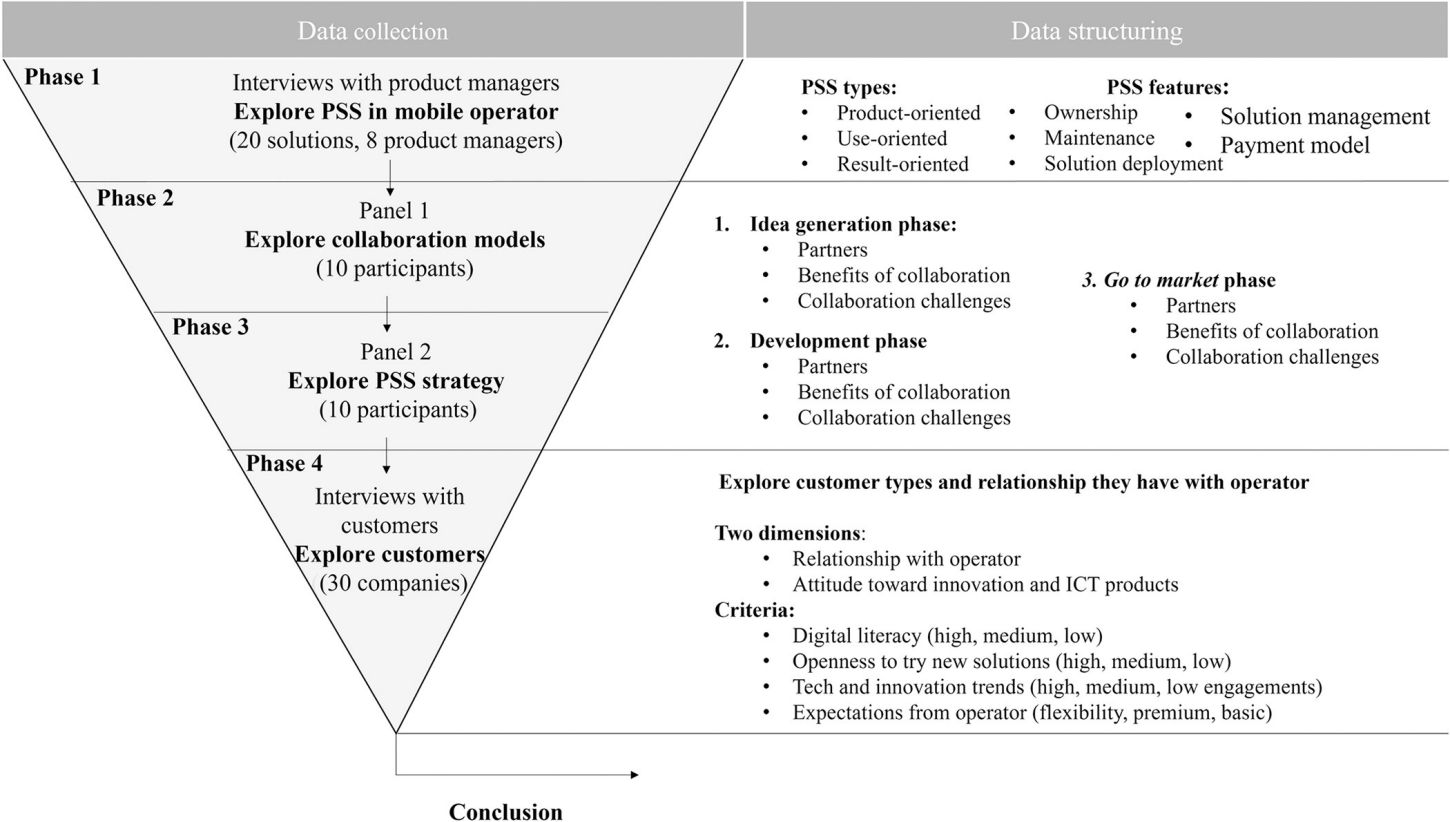
Research methodology.

The research is conducted in a private telco operator. The company is a member of an international group which has over 26 million customers across seven countries in Europe. Case study consists of four phases. To provide a comprehensive picture of the study, the data were gathered from a variety of sources including employees from the company (experts and decision makers), documents from the organization and customers (the end users of services provided by telco operator), which enabled triangulation of data and validation of study [74]. A case study protocol and database were created in order to improve reliability of the study. The research methodology is presented in Fig 2.

**Phase 1**–20 solutions and projects that are combination of products and services are selected and interviews with 8 product managers conducted. The purpose of interviews was to investigate the practice regarding the collaboration with different partners and PSS business models. Experts have more than five years of experience in building operator portfolios. Interviews lasted around 1.5 hours. The respondents were asked open questions with the support of an interview guide. Data from discussions are complemented and validated with other materials such as documentations, manuals, and product presentations.

**Phase 2** and **Phase** 3 were two panel discussions. 10 experts and decision makers participated in each panel: 6 product managers, manager of product development team, manager of presales team, sales director, and ICT solutions director. Participants were encouraged to debate, exchange ideas, and reach common understanding about the topics discussed. One panel discussion was about products and services, problems, and examples of good practice in the development and *go to market* phase regarding partnerships. The second panel discussion was about the strategic approach toward collaborative servitization. The study findings were presented back to the company to get additional comments and to reduce the influence of subjectivity introduced by the study method. Additionally, secondary data (organization documentation) were used to triangulate findings, mitigate method bias and improve validity [74].

**Phase 4**–30 different companies (business customers, one representative from each company) filled in a questionnaire about their expectations from telecommunication providers and their services, their innovativeness and interest in trying new technologies.

Participants were informed about the purpose of the research and the use of data they provide. The authors did not ask the respondents for any confidential information and the data used in this study are completely anonymized.

## 4. Results

### 4.1. PSS in mobile operator company- Portfolio analysis

The starting point of the research is to investigate operator current portfolio and the solutions in the context of servitization. Twenty different solutions that are combination of products (hardware and software) and services (connectivity, consultancy, maintenance, reports…) are analyzed. Solutions are divided into three groups:

1. Mass market IoT solutions (IoT connectivity, IoT platform, smart home solution, smart cash register, vehicle tracking system)
2. Tailored made IoT solutions (complex IoT solutions for monitoring, automation and controlling of different processes and devices. Solutions are executed as a project, specifically created to respond to customer business and requirements. Some projects were done in agriculture, some in production processes and logistics (due to privacy, authors cannot write the full specification of projects).
3. Other ICT products (cloud services (data hosting, cloud probate branch exchange (pbx), backup as a service) and solutions related to infrastructure and network (telehousing, software-defined wide area network), printing as a service, Microsoft services).

The business model can vary depending on the end customers wishes and the product itself. What is common for all solutions is that maintenance and the initial set up of the equipment are usually included in the price. Most of the solutions involve SIM cards, and the voice and data usages as monthly fees. Hardware is sold eighter as a fixed price product at the beginning of the contract, or it is paid monthly through the bill. In these cases, the customers are the owners of devices.

**Fig 3:**
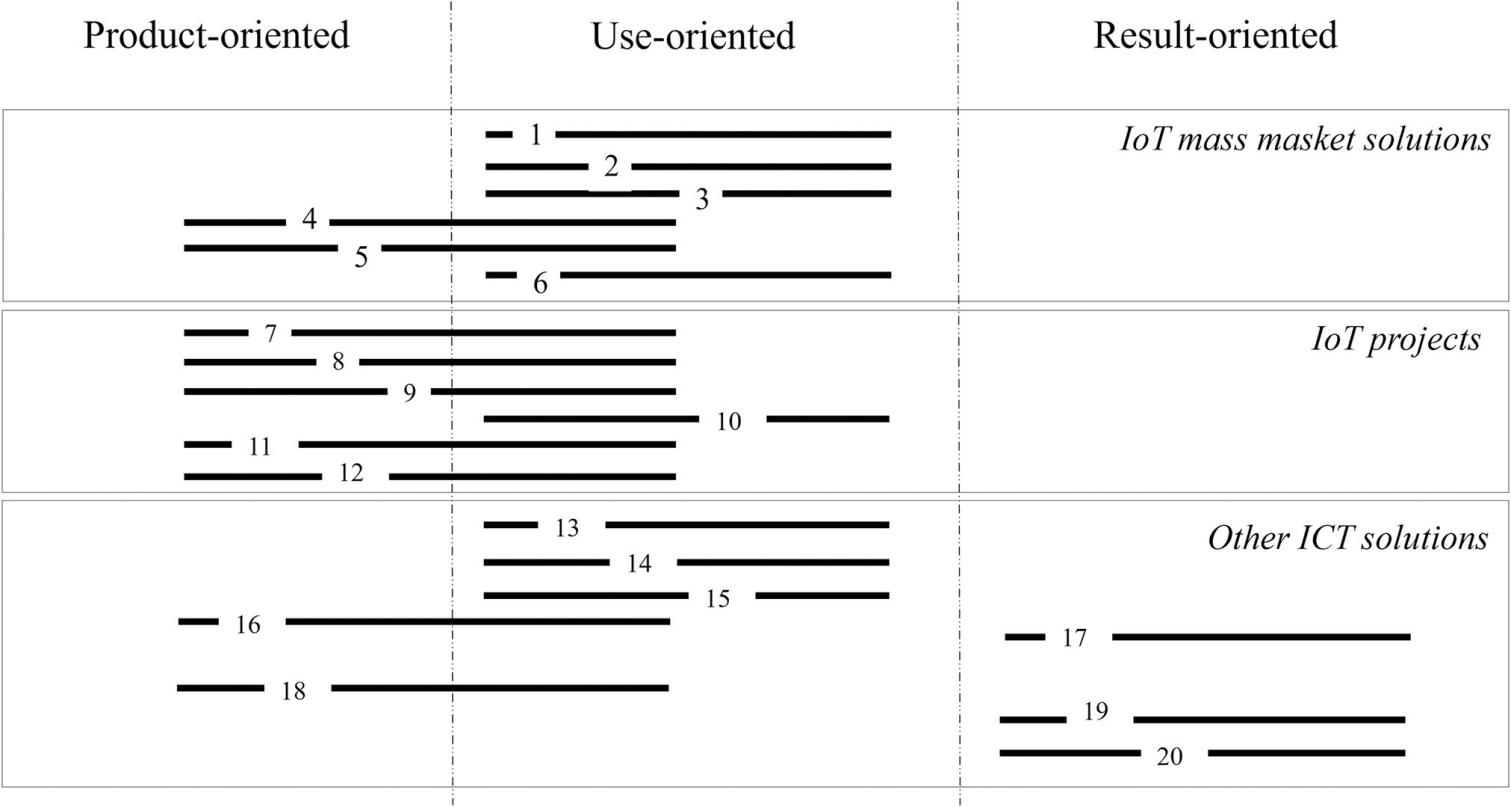
The analyzed solutions based on type of PSS (product-oriented, use-oriented, result-oriented).

A mixture of product-oriented and use-oriented solution is when hardware is sold as a one-time fee at the beginning of the contract, and then customers pay fixed monthly fee for using the services (8 solutions). In some cases, the price of HW is bundled with a fixed monthly service fee, so the customers only pay fixed monthly fees during the contract (4 solutions). Another type of use-oriented solutions are licensed-based products, which means customers get a service and pay monthly fee for using it (4 solutions). In the operator portfolio, there are also examples of result-oriented products (3 solutions). Fig 3 illustrates the analyzed solutions in a matrix format, with two dimensions representing the business model types (product-oriented, use-oriented and result-oriented) and solution types (mass market IoT solution, tailored made IoT solution, and other ICT products).

As it can be seen, Table 1 presents the features of the observed solutions. First level support for solutions is always done by the operator. According to the service level agreement (SLA) operator should solve any problem that occurs with product. Second level support is done by operator or partner, and third level support is, in most cases, done by partners and hence strong SLA with partners is important to ensure successful incident solving.

**Table 1:** PSS features in the case study.

Feature	Option	Frequency
Ownership	Customer	13 (65%)
	Operator	7 (35%)
Maintenance	Customer	0
	Operator	20 (100%)
Solution deployment	Customer	6 (30%)
	Operator	14 (70%)
Solution management	Customer	14 (70%)
	Operator	6 (30%)
Payment model	One time at the beginning	0 (0%)
	One time + monthly rate	8 (40%)
	Fixed monthly rate	5 (25%)
	License based	4 (20%)
	Pay per use	3i(15%)

Analyzed solutions are all developed with at least one partner (except two solutions that were created internally). 11 solutions are developed with one partner, and 7 with two or more partners (4 solutions with two partners, and 3 solutions with three partners) (Table 2).

**Table 2:** Collaboration with partners in different stages.

**Solution development phase**	**Frequency**
Internal	2 (10%)
One partner	11 (55%)
Two and more partners	7 (35%)
***Go to market* phase**	**Frequency**
Only internal sales force	16 (80%)
Internal sales and partners	4 (20%)

*Go to market* phase, is challenging by nature, even more if the market is unknown for company. Mobile operator as a connectivity provider is not seen as a solution provider, for example in the field of IoT or in the field of security. Firstly, people are not aware of all the services the operator has. Secondly, even if customers knew about the services, they would question the operator’s competences. In those cases, partners can help the operator establish the customer relationship and gain the necessary trust.

In this research 5 out of 20 solutions have at least one partner that is selling the solution, and there is a plan to start selling three more products through the partner network.

### 4.2. Collaborative servitization in mobile operator company

The study investigates three phases of PSS development within the context of collaboration: idea generation, where operator and partner companies collaborate during the search for new ideas, conduct market research, and validate concepts; development, where partner companies and operator collaborate in creating the solution; and go-to-market, where partner companies and operator collaborate during the product’s commercialization.

Benefits and challenges of collaboration with partners are observed through three aspects:

- Solution related (benefits and challenges related to new bundles of products and services)
- Relationship related (focused on relationship with partners, communication, the complexity of collaboration…)
- Business related (how certain situations impact operator costs and the time)

Results are presented in Table 3.

**Table 3:** Collaborative servitization—benefits and challenges.

	Idea generation phase	Development phase	*Go to market* phase
**Partners**	• Customers • Consultants • Innovation hubs • Startups	• Customers • System integrators • Suppliers • Startups • Universities	• Friendly customers • Experienced companies • Recognized individuals
**Benefits of collaboration**	**SOLUTION FACTORS** • Learn about new solutions (products and services) and technologies that aren’t operator main business • Identify new business opportunities (outside of operator competencies) • Consultancy regarding the idea validation **RELATIONSHIP FACTORS** • Proximity with partners from other domains leads to generating common ideas about new solutions • Being part of startup community opens possibility to discover different solutions	**SOLUTION FACTORS** • Develop new solutions where there is lack of competences and domain knowledge • Expand portfolio toward more complex solutions • Enable small modifications (Customization of standard products) • Adequate extension to standard products **RELATIONSHIP FACTORS** • Have a pool of reliable partners that can quickly respond to requirements **BUSINESS FACTORS (TIME AND COST)** • Quickly respond to customer needs	**SOLUTION FACTORS** • Raise awareness in the new market (inform customers about new portfolio) • Reach new customers through recognized partners in the domain • Position on the market (increase market share) **RELATIONSHIP FACTORS** • With the increase of number of partners complexity of collaboration is not changed • Find a partner that will offer solution to the customer when he needs it *Product Manager*: *“For smart home we created partnership with insurance companies*. *When a customer thinks about ensuring an apartment*, *he thinks about the security of his home*, *and then considers buying smart home devices*. *When a customer enters our shop*, *he doesn’t think about home security”*.
**Collaboration challenges**	**SOLUTION FACTORS** • The niche solutions that don’t have many potential customers are not profitable • If niche solution is developed there must be a big project (in terms of investment) behind otherwise it doesn’t pay off **RELATIONSHIP FACTORS** • Working with startups can be risky due to their immaturity	**SOLUTION FACTORS** • The success of solution depends on all partners not only operator *Product manager*: „*We are the only contact with customers and we are responsible for solution excellence*. *But we don’t control every aspect of the solution and we depend on our partners*. *Partner fault is our fault toward customers“*. **RELATIONSHIP FACTORS** • Complexity increases with the increase of number of partners • When working with more than 2 partners communication between partners is harder, agility is hard to reach, and responsiveness is lower *Product manager*: “*On one project we worked with three successful companies on developing a complex IoT solution*. *Organizing a quick meeting was almost impossible*, *we always had to define several days in advance the date of the meeting*. *Project was canceled at the end*.*”* • It is harder to identify problem (responsibility) and clear SLA procedure is needed *Product manager*: *Customer experience and solution excellence is of great importance*, *so in the case of incident or customer complaint it can be hard to determine on whose side is the problem*, *so a detailed and clear reasonability matrix and SLA are needed*. **BUSINESS FACTORS (TIME AND COST)** • Operator doesn’t understand the hardware business (time needed to purchase components), and can wrongly estimate the development time	**RELATIONSHIP FACTORS** • There should be a clear procedure for partner onboarding *Solutions director*: “*We have partners that sell out products*, *however*, *we don’t have clear structure for partner selection and onboarding and that is why the process to sell product through partner can take a lot of time*. *We need to dedicate a person who will lead all the processes and be responsible for all the activities strategically and in execution”*. *Sales director*: “*Not having a clear structure for partner selection results in a long onboarding process*. *We had a simple product for selling through partner network but distributing it through partner network took as much time as it took us to develop solution because we didn’t have a strategy*”.

A first step in creating new bundles of products and services is to identify the customer needs, and to agree on high level design of a solution. Sources for new ideas are conferences, reports, other operators, and their portfolios, but also customers, their needs and wishes. What is common for all ideas is that they go through testing phase (validation phase) with customers. Innovation hubs and consultant companies help with market research and testing. They organize interviews as focus groups or scheduled meetings with customers, or provide, if exist, some statistical reports. In some cases, the operator takes advantage of the existing customer base, and if possible, gets feedback from his customers about their interest in new solutions.

Sometimes startups reach operator with their idea they work on, they need help to find customers, or they need a partner for developing a solution. 40% of experts during the panel discussion believe that working with startup is too risky, however they all agreed that operator should be closed to startup ecosystem, and monitor activities they have, in the case some good opportunity appears.

The operator tends to create a network of reliable partners, with whom they can cooperate on several projects. Complex projects need several partners. Even though the responsibility is clearly divided, communication is harder, it takes longer time to agree on the meeting time, and it takes more time to get feedback from all sides. Agility and responsiveness tend to be lost in projects that involve several experts from different companies.

During the first panel discussion, experts agreed that projects that involved one partner were easier to manage and had more success. Projects involving two partners were also successful but did experience some difficulties in communication and clear responsibility sharing. Projects involving more than two partners were hard to handle, and some were not successful, especially due to lack of agility and longer response time, it was hard to coordinate meetings and schedule quick meetings.

During the second panel discussion experts pointed out the importance of well-defined SLA and incident management platform. Customer experience and solution excellence is of great importance, so in the case of an incident or customer complaint, it can be hard to determine who is responsible for the problem. Hence clear procedures and a responsibility matrix needs to exist. The operator doesn’t control the whole solution and toward the customers he guarantees excellence, therefore there is a risk of failure due to his partner’s faults.

During the project companies get to know each other better and understand each other’s capabilities, knowledge, expertise, and way of working. The experts agreed that if one project was successful, there is a high probability that companies will cooperate again. When the operator knows partner, he knows what to expect. Additionally, if he doesn’t need to search for partner, he can get quicker response to customer demand. However, 60% of panel participants agreed that if there is time, there should be a partner (vendor) selection process to get a better price and to leave the possibility of finding and testing some potentially new partners.

In the *go to market* phase, it is challenging to reach customers, and to convince them the solution is good, and that the operator is a reliable solution provider. Partnerships with recognized experts in the domain can be of help. One example is a product for smart homes. These sensors were part of the operator portfolio for several years, however, there was not much success in selling. Then the partnership with insurance companies was signed. Insurance companies were selling a smart home solution together with home insurance policy and they managed to sell three times more devices than operator. Similar situation was repeated with device that measured temperature. Transportation companies were not interested in investing in this device for remote monitoring of temperature in vehicles transporting temperature sensitive goods, since they were covered by insurance. However, insurance companies found this product very useful for combining it with insurance policies for transportation.

The operator can partner up with recognized individuals in the several industries and use their reputation to promote themselves. All participants of the second panel agreed that operators need different *go to market* strategies. One proposed solution was mediator, a recognized individual in the public, that can help operator reach customers, to establish a good reputation and become recognized as a solution provider.

Simple solutions are offered through a partner network. For a specific product, the operator can have several partners. If there is a collaboration model defined, the complexity doesn’t increase with having more partners for the same solutions (like it is the case in solution design). During the panel discussion experts agreed that gaining customers through a partner is challenging and requires a procedure for partner finding, evaluation and onboarding. They also agreed that there should be a dedicated person who leads all the processes for partner onboarding and is responsible for all the activities strategically and in execution.

#### 4.2.1. Customer participation in the development of PSS

The customer, as a partner in developing PSS, is addressed separately and the main results are presented in Table 4.

**Table 4:** Collaborative servitization with customer as a partner.

	Idea generation phase	Development phase	*Go to market* phase
**Benefits of collaboration**	**SOLUTION FACTORS** • Understand the industry • Identify what are customer needs and what they are willing to pay the money for • Create concept for optimal solutions *Product manager*:” Solution *should measure what customer needs are and how frequently customers need information*. *Investing in solution without having customers doesn’t pay off*, *so we wait for customers to come to us*, *and then to create a specific solution according to their requirements*.*”* **RELATIONSHIP FACTORS** • Establish a partner relationship from the early stage of PSS development **BUSINESS FACTORS (TIME AND COST)** • Design a solution that is optimal in terms of price, functionalities and customer needs	**SOLUTION FACTORS** • Understand the need and approach it in the right way • Modify solution in the early stage of development • Improving standard services • Understand the industry and way to approach it Product Manager: “*Customers pay for connectivity services once a month*, *but it turned out several customers were interested in paying once a year*, *or even to pay at once for three years*, *so we created product*, *that made this possible*.*”* **RELATIONSHIP FACTORS** • Proof of concept is a method to get valuable feedback from customers, and for customers an opportunity to understand the solution and the value it brings **BUSINESS FACTORS (TIME AND COST)** • If some changes and corrections need to be made on the solution, they will be identified before going to market phase and, in such way, save the money and solution from failure	**SOLUTION FACTORS** • Friendly customers can be used as good reference on the market • Friendly customers are the first to use solution and as such can be used in marketing purposes • Through friendly customers operators position in the community where friendly customers belong
**Collaboration challenges**	**SOLUTION FACTORS** • Not to customize solution too much • If niche solution is developed there must be a big project (in terms of investment) behind otherwise it doesn’t pay off **RELATIONSHIP FACTORS** • Misunderstand customers **BUSINESS FACTORS (TIME AND COST)** • It takes a lot of time to get to final solution (in terms of generating revenue) • Too much customization doesn’t pay off in terms of investment and time needed to develop solution	**SOLUTION FACTORS** • Too much customization and unnecessary requirements that might lead to project failure **RELATIONSHIP FACTORS** • If customers are not much involved in the project it can last longer than planned, the feedback might not be adequate • Negligent equipment testing *Product manager*: *„During one project customer lost the devices during testing*. *Since it was just a test period he got it for free and he was careless about the equipment*.*“* **BUSINESS FACTORS (TIME AND COST)** • Too many requirements result in unnecessary extension of the project • Too much customization might result in significant price increase that customers are not willing to pay	**BUSINESS FACTORS (TIME AND COST)** • Friendly customers use solution for free, or with significantly lower margin

All the experts, during the panel discussion, agreed that customers are valuable resources for new ideas. However, not all of them agreed that starting from customer ideas is a good strategy, 20% percent of them find it too slow (in terms of generating revenue) and too narrow (in terms of mass production).

In the IoT ecosystem complex solutions are designed for specific customer requirements and customer demands are usually triggers for new solutions. This is because the design of the solution depends on the information customers need, how frequently they need data, where are the devices situated, etc.

Experts agreed that it is important to relate the idea to the existing strategy. Moreover, the solution should be profitable eighter by number of devices ordered by single customer or solution has a wide market and can be used by other companies.

The panelists agreed that proof-of-concept (PoC) is a good method to get valuable feedback from customers, and to introduce a solution to customers. They also pointed out that PoC should be conducted with strategically selected companies that have a potential to become good promoters of solutions in the future. However, if customers are not much involved (interested) in the project PoC might be unsuccessful.

An example is a project in which a customer lost testing devices (they fell from the wall of container and were never found again).

Friendly customers can help with the commercialization of the product. When a product is new on the market, the operator offers some special promotions, for his first users. This can be beneficial for both customers and operator. The operator can use references and successful stories from his first customers in marketing purposes to increase sale of a solution, and customers can benefit in price. For some complex solutions the operator offers a free trial period for customers to test and decide upon buying a solution (an example is SD-WAN solution).

Panelists agreed that customers are important partners in all three observed phases, but they also pointed out that not all cooperations were successful and that customers should be carefully selected. To explore the potential of customer participation in the development of PSS, 30 companies (users of different telecommunication services) were questioned (one representative from each company).

**Fig 4:**
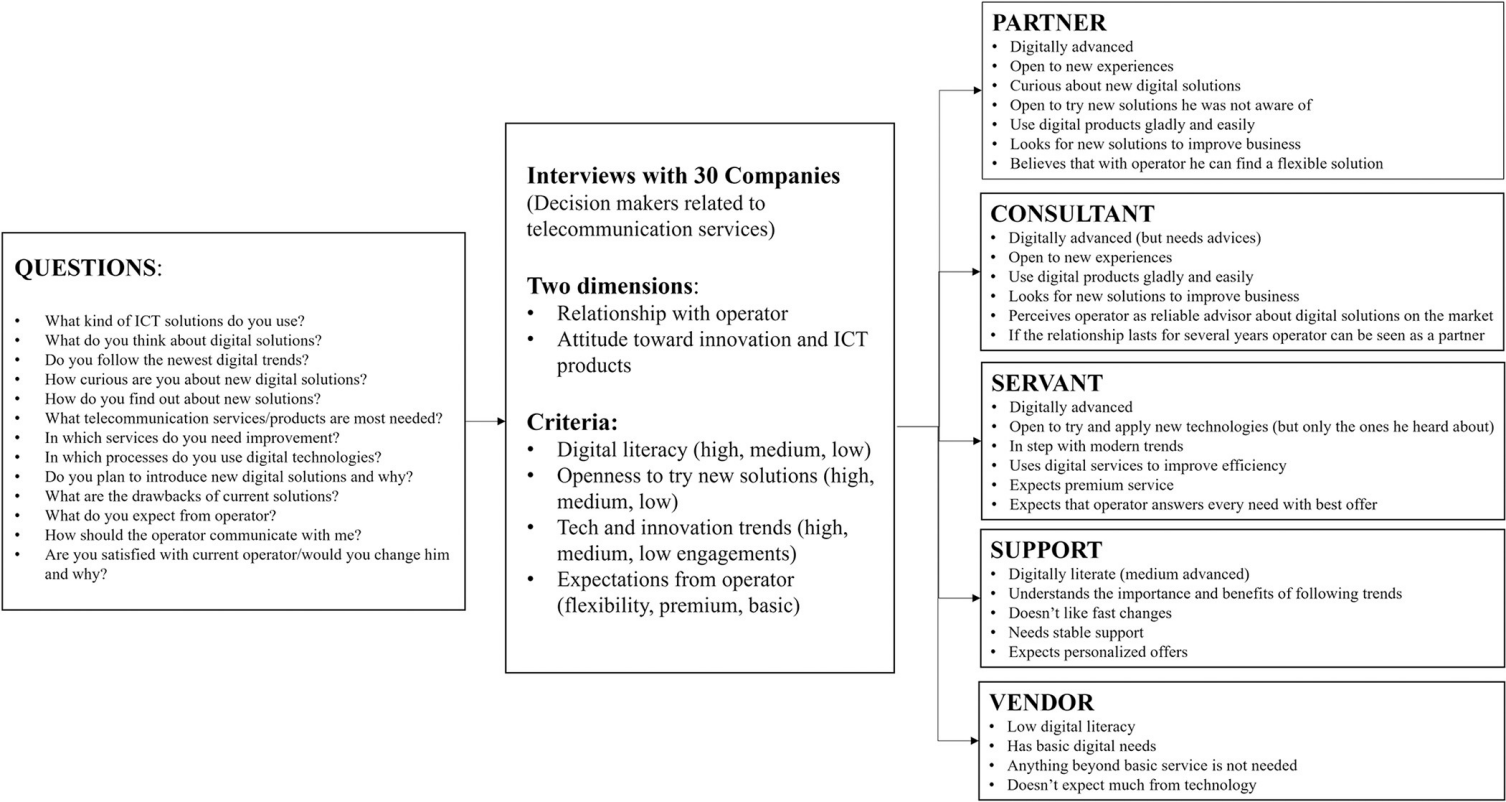
Different customer types.

**Fig 5:**
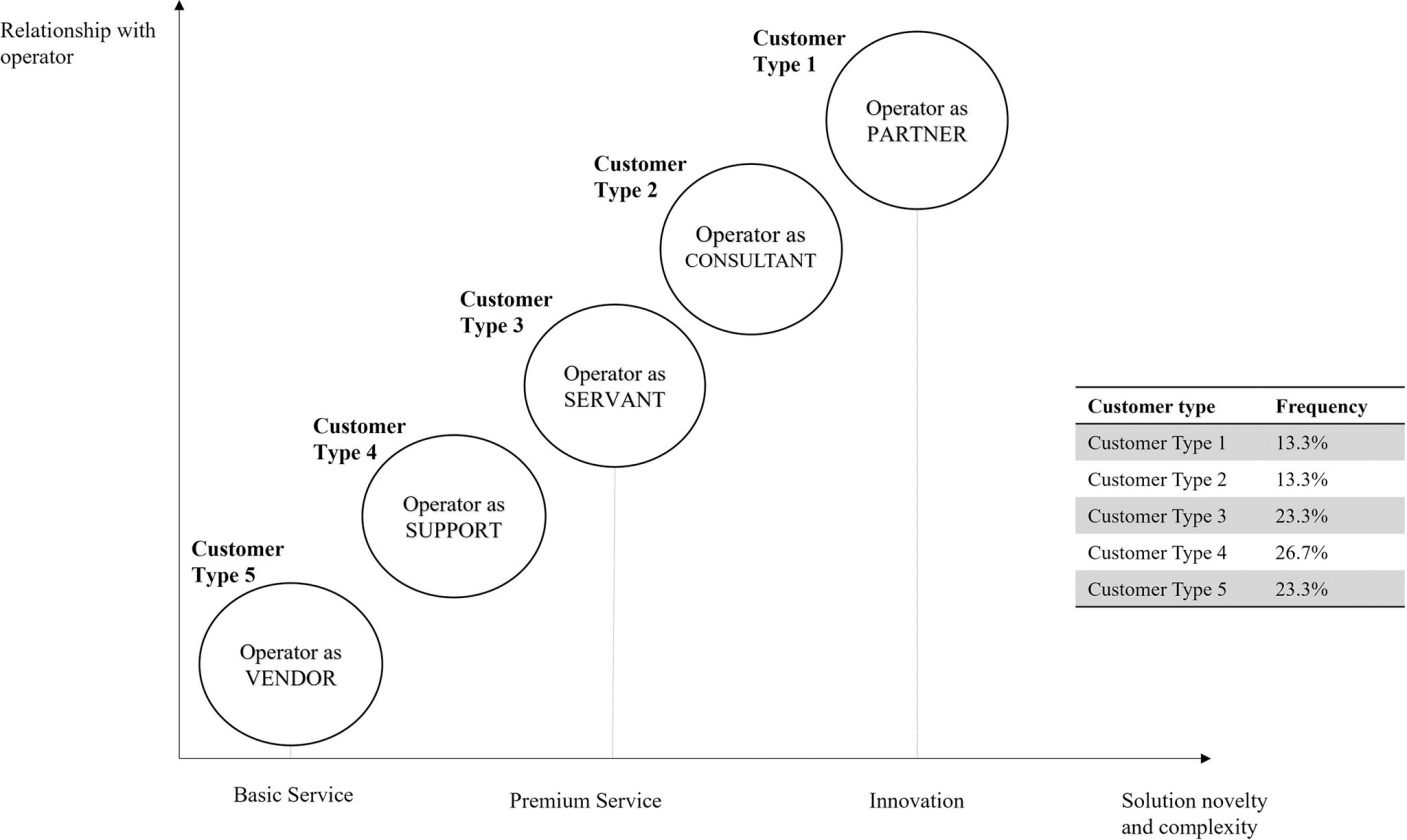
Customer types according to toward digital solutions and the relationship with operator.

Based on the answers customers provided five different customer profiles are identified. The profiles differ in the attitude customers have toward digital solutions (innovation and technology) and the relationship they have with operator (Figs 4 and 5).

One of the profiles (customer type 1) is specifically open to new experiences and innovation. Customers from this segment see telco operator as a partner, they are digitally advanced and in step with modern trends, easily adopting and implementing innovations and generally open to new experiences. Customers belonging to this type are open to trying and accepting solutions they were not aware they needed. As such, these customers are convenient for testing new solutions. In this research, customer type 1 was 13% of the total number of participants.

The two other customer profiles (type 2 and type 3) are also technologically advanced. One group perceives operator as a consultant and needs operator for improving business (but with existing and verified solutions). Customers from the third segment want and pay only for the premium service, they follow the newest digital trends. They are not suitable for being a partner in innovation, because developing new products means learning from mistakes and being flexible for modifications, and this group is exact and doesn’t tolerate mistakes.

Other profiles are less digitally literate (type 4 and type 5). One customer group is aware of the importance of digital modern trends but is more a follower. This group sees telecommunication provider as support that gives help and advice. The least digitally advanced customer group perceives telco provider as vendor and doesn’t need anything beyond basic service.

## 5. Discussion

The case study has shown that PSS is well established in operator’s portfolio. There are different business models, depending on ownership, solution management and payment model. These features define a mixture of product-oriented, use-oriented, and result-oriented servitization models.

For most of the complex ICT solutions mobile operators rely on partners since they don’t possess all relevant capabilities and resources for developing and exploiting PSS to customers. Existing research on collaborative servitization emphasize the need for more practical, micro level studies to understand collaborative practices [2, 17, 56]. Some analogies were found with previous studies [38–40] that suggested different approaches to servitization depending on phase: idea generation, development and go to market phase. However, this research, by focusing only on telco industry explores deeper the collaboration practice in each of the phases and identifies partners, benefits and challenges of collaboration and gives real-life examples. In each of the analyzed stages the mobile operator collaborates with different partner, in a different way and with a different goal.

Given that the first research question was how companies benefit from collaboration models in different phases of PSS and what are the challenges, benefits and challenges were observed through 3 aspects (to organize and synthesize results in an understandable way): one associated to solutions (product and services as integrated packages), one group presenting relationship with partners and one showing how collaborative servitization impacts operator time and costs (Table 3 summarizes the results of collaborative practises with different partners while Table 4 focuses only on customer as a partner).

In the idea generation phase customers play the most important role, since their requirements and problems are taken as a base for new products and services. Solution is constructed based on customer needs for services, and the offer can be packed according to requests. Innovation hubs and consultants sometimes navigate the process of testing an idea. Ideas for new solutions might also come from outside of the organization, when smaller companies (usually startups) seek for partner for scaling up their business. During the development phase, collaboration is established between the operator and companies who have knowledge to develop a solution: system integrators, startups, suppliers, universities. In *go to market* phase collaboration is established with companies and individuals that are respected in the field where solution is positioned, or with companies that can reach customers with the solution when customers need them.

Each phase poses different challenges in terms of collaboration (relationship related factors). For example, the increase in the number of partners increases complexity in the development phase (agility is hard to reach, it is hard to coordinate everyone’s availability, or to identify responsibility for solution malfunctioning and clear SLA and incident management procedure is crucial). On the other hand, in *go to market* phase for one solution operator may have many partners, and the number of partners is not increasing the complexity of the model. However, in the *go to market* phase special models and collaboration principles for partner selection and partner onboarding need to be created to be effective and productive in selling across partner network.

With the right partners collaboration models can bring satisfactory results in each of the analyzed phases, firstly to identify the right solution, develop it according to requirements and monetize it on the market which proves that partnerships are very important for successful application of PSS in service-oriented companies. Special attention should be given to challenges of collaboration in the context of complexity in communication and responsibility sharing.

Next research question was investigating the importance of customer involvement in PSS development, and it identifies benefits and challenges of that collaboration. The study confirms previous research from manufacturing industry [1, 22, 23] about significance of customer participation in all phases of PSS development. As can be seen in Table 4, during idea generation solution is designed according to customer feedback, and each new idea is tested with customers before going into development. In the development phase, customer feedback during PoC is of crucial importance for later project realization. Collaborating with customers allows operators to minimize failure in the sense of creating a solution that customers need and are willing to pay for. During the *go to market* phase, friendly customers can be of great help for product promotion. Customer capabilities are important for solution success, as shown in [24], the panelists agreed that not all customers are interested in participating in PSS development, testing, and providing feedback. To investigate this more thoroughly, 30 business customers filled in a survey, and the research has shown that only one group of customers (among 5 that were identified in the research) perceives operator as a partner in business and is open to innovate and try new solutions (solutions that possibly can positively impact their business). Potentially, another customer type that sees an operator as a consultant could be a suitable group for friendly customers. Overall results lead to the conclusion that customers (when carefully selected) play important role in the implementation of PSS.

Previous studies emphasized the importance of strategic approach toward collaborative servitization [26, 62] and how projects fail when solutions don’t create sufficient value compared to costs they are causing [22]. To better understand this, in this study the strategic aspects are analysed in each stage of PSS development as a third research topic. The panel participants fully agreed that collaboration models are matter of strategic principles. For example, as shown in Table 4 under business aspects, in the idea generation and development phase operator should balance between the number of requirements and customer satisfaction. Requirements should be limited since too much customization results in price increase and realization time increase, and that might not be satisfactory for customer. Niche solutions are not mass-market solutions, and their profitability is questionable, but if a solution is created through a big project, it has potential to be successful in terms of investment.

In addition, operator balances the trade-off between complexity of the solution and complexity of relationship with partners. Operator is responsible for solution performance toward customers and with more partners the risk of failure is more dependent to third party (relationship factors in Table 3).

Moreover, having partner in *go to market* phase means having a lower margin (the collaboration model is seen as an investment for creating a successful and well-established position on the market), so operator strategically selects partners.

Finally, customers are of great value in collaborative servitization, but they need to be carefully selected (according to customer types) and their requirements should match company’s strategy and solution itself should be profitable in terms of the number of potential devices per single customer, or in terms of the size of the market (the number of customers that might use solution).

## 6. Conclusion

Telecommunication companies are expanding their scope of services with integrated bundles of products and services to ensure growth in their own market but also to expand to other markers due to the digitalization trends. Because of changes in the telco sector, the lack of competence and lack of experience in the new markets mobile operators tend to establish different collaboration practices that they incorporate into specialized offers for their customers. Through a case study in an international telco operator company, it was shown that PSS and the role of partnerships in the context of servitization, are of strategic importance in idea generation, development and in *go to market* phase. Each phase demands a different approach and different partners, while the customers are regarded as important partners in all the stages.

Previous studies have primarily focused on exploring collaborative servitization within manufacturing companies without specifically analyzing the growth of service companies in applying PSS. The study contributes to the research on collaborative servitization by taking a different approach and observing collaborative servitization in companies that added products to services.

The findings of the paper offer concrete inputs about collaborative servitization: who are the partners, what are the benefits and the challenges regarding solutions, relationship with partners, time, and costs in different stages of PSS development. Several managerial implications can be drawn for telco companies that move toward PSS or need improvements in application of collaborative servitization. Firstly, by indicating benefits and challenges of collaborative servitization in different phases. Secondly by pointing out the importance of collaborating with customers. The research identified different customer types, and only one group that is suitable for the role of partner. Finally, the study highlights strategic aspect that is different in each stage of PSS development, and it can help managers understand their role and its importance in successful PSS application.

The present study has certain limitations that should be considered when interpreting the outcomes. One of the limitations of the study is observing telco industry in general while the study focus on specific segments of the mobile industry meaning mobile operators. Producers of telco equipment are more like manufacturers, and it would be interesting to conduct study in other telco segments such as system integrators, equipment producers in the future and compare it with this one. Secondly, study was more focused on PSS development and go to market strategy for PSS, rather than PSS operations. Additionally, the servitization was addressed only in business-to-business context (B2B). Finally, the analysis of 30 companies has shown that only certain customer types are eligible for the role of the partner, but it did not provide an answer how big is that group (compared to other groups also identified). The next step would be to translate these finding to a larger scale research to obtain more general approach how to identify, select and onboard customers as partners in development of PSS.

## Supporting information

S1 File (XLSX)
